# Supplementary material for: The frequency and importance of polyploidy in tropical rainforest tree radiations
Source: New Phytol. 2025 Nov 23;249(4):2103–14. doi: 10.1111/nph.70764 (PMC12824605; doi:10.1111/nph.70764)
Supplement: Supplementary file 1 — Fig. S1 nQuack allele frequency histograms for putative tetraploids. Fig. S2 Boxplot showing number of defence chemistry loci under selection for each ploidy. [file NPH-249-2103-s001.pdf]

New Phytologist

New Phytologist Supporting Information 1

Article title:
The frequency and importance of polyploidy in tropical rainforest tree radiations

Authors: Rowan J. Schley, Rosalía Piñeiro, James A. Nicholls, Michelle Gaynor, Gwilym P. Lewis, Flávia Pezzini, Kyle G. Dexter, Catherine Kidner, R. Toby Pennington, Alex D. Twyford

Article acceptance date: 29 October 2025

<u>The following Supporting Information is available for this article:</u>

Fig. S1
nQuack allele frequency histograms for putative tetraploids

Fig. S2
Boxplot showing number of defence chemistry loci under selection for each ploidy

Table S1
Ploidy inference and sample information

Table S2
Taxa included in BUSTED analyses: polyploids and six sets of diploids

Table S3
Ploidy phylogenetic signal modelling results

Table S4
Counts of loci under selection for each BUSTED ploidy run

Table S5
BUSTED selection $\chi 2$ results: selection x annotation type for each ploidy level

Table S6
BUSTED selection $\chi 2$ results: selection x ploidy for each annotation type

Supplementary figures

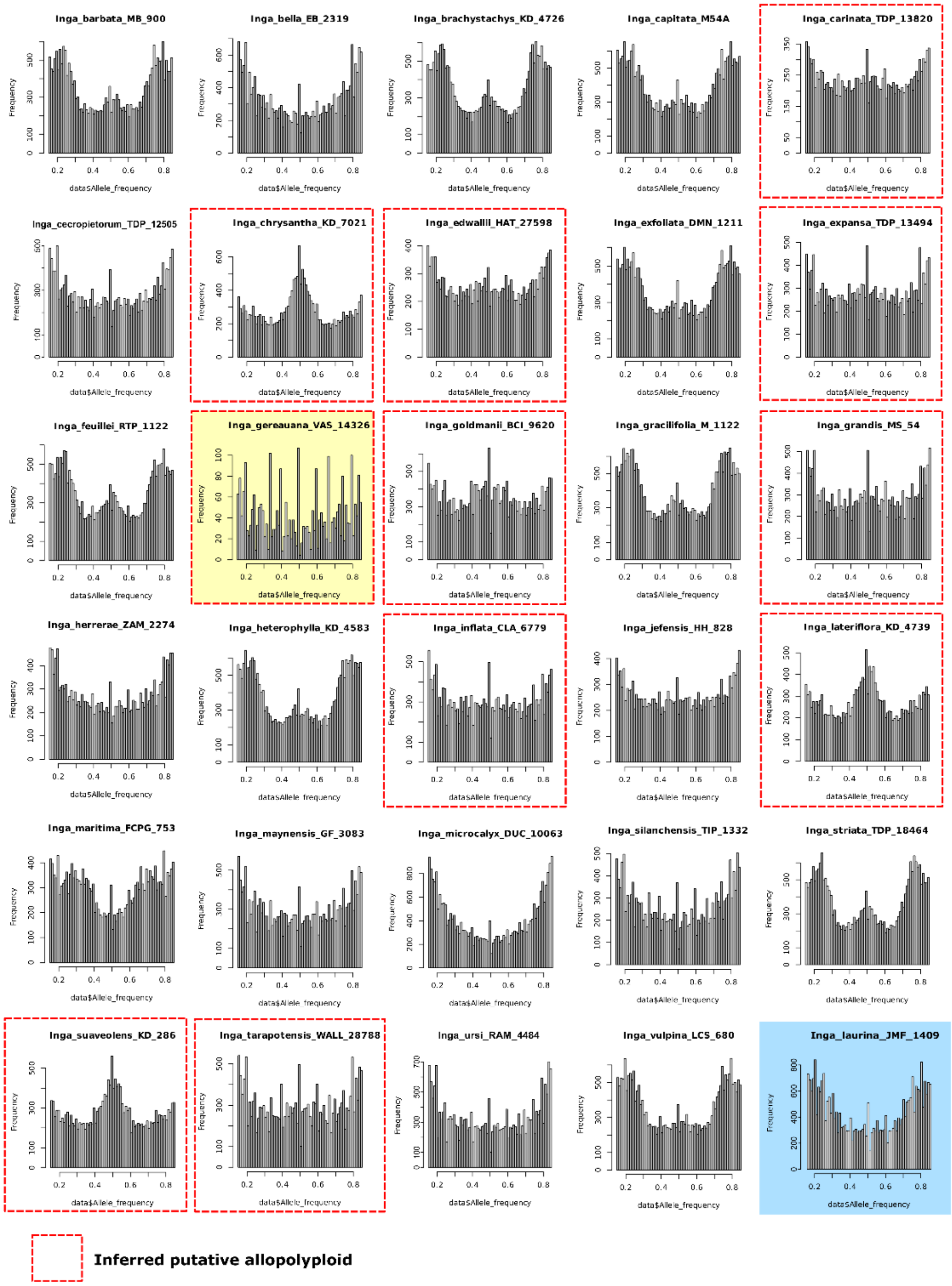

Figure S1: nQuack allele frequency histograms for accessions inferred to be tetraploids using allele frequency ratios in nQuack. Species with elevated alpha values (i.e. proportions of sites) at an allele frequency of around 0.5 (corresponding to a tetraploid genotype of aabb) relative to the rest of the histogram were inferred to be putative allotetraploids (i.e. contain two divergent subgenomes) – these putative allotetraploids are marked with a red dashed box. *Inga_gereuana_VAS_14326*, which had low locus recovery and poor sequency quality in previous work (Schley *et al.,* 2025) and a noisy allele frequency histogram in this study is indicated by a red dashed box with a yellow centre. Marked in blue is the newly sequenced accession from this study for *Inga laurina* from Brazil, which was tetraploid, whereas its conspecific *Inga_laurina* TI_1387 was diploid.

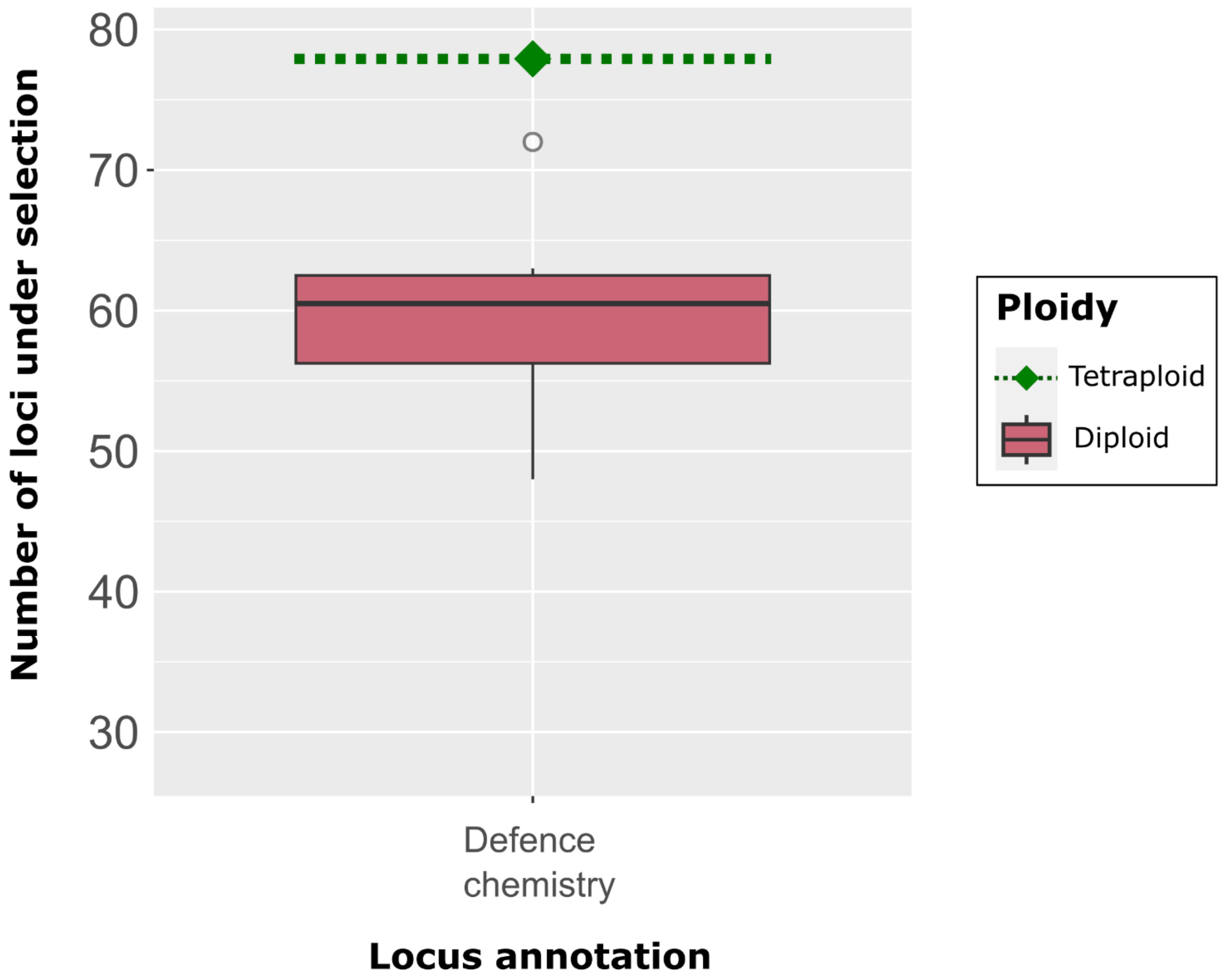

Figure S2: Box plots indicating the numbers of *Inga* HybSeq loci underlying chemical defence against herbivory that were inferred to be under selection by BUSTED ('under selection' if false-discovery-rate-corrected P value <0.05). Displayed in the plot are the counts of loci inferred to be under selection from the ‘tetraploid’ BUSTED run, as well as the six randomised ‘diploid’ BUSTED runs, all of which comprised ca. 28 taxa. The x-axis indicates the target capture locus annotation and bar colours indicate whether each counted locus was from the ‘polyploid’ or ‘diploid’ BUSTED run. For the diploid box, the dark bar represents the median, the top and bottom edges of each box represent the first and third quartiles, while the white circles represent outliers. The tetraploid point and dotted line represents the number of loci under selection from the single polyploid BUSTED run.

Counts of states	*Diploid* = 161<br>*Tetraploid* = 29
Number of permutations	10000
Estimated D	0.876
Probability of E(D) resulting from no (random) phylogenetic structure	0.347
Probability of E(D) resulting from Brownian phylogenetic structure	0.070

Table S3: Model output from the Phylo.D function of Fritz and Purvis (2010), used to calculate phylogenetic signal of polyploidy across the *Inga* phylogenetic tree. From top to bottom, rows indicate the counts of both character states across the *Inga* phylogenetic tree, the number of permutations used to generate a null expectation, estimated D values indicating phylogenetic signal and, finally, the probability (P-values) of attaining the observed D value under two null hypotheses – one under a model of random trait evolution, and the other under a model where the trait evolves via Brownian motion.

	N species in BUSTED run	N loci under selection				% total loci under selection
		Defence chemistry	*Differentially expressed*	*Miscellaneous*	*Single-copy phylo.*	
Diploid set 1	27	48	33	22	427	43.91% (*530*)
Diploid set 2	27	60	38	20	478	49.378% (*596*)
Diploid set 3	27	61	42	29	498	52.195% (*630*)
Diploid set 4	27	72	56	29	592	62.054% (*749*)
Diploid set 5	27	55	44	22	488	50.455% (*609*)
Diploid set 6	28	63	54	20	498	52.609% (*635*)
**Tetraploid**	**29**	**78**	**60**	**30**	**627**	**65.866% (*795*)**

Table S4: Counts of Mimobaits loci under selection (i.e. with false-discovery-rate-corrected BUSTED P-value <0.05) for each BUSTED run: six runs of ca. 28 diploid *Inga* species, and one with only polyploid *Inga* species. Selection results are parsed by Mimobaits locus annotation type ('Defence Chemistry', 'Differentially expressed', 'Miscellaneous' and 'Single-copy phylogenetically informative' loci). In the final column, the number (in parentheses) and percentage of all loci inferred to be under selection is shown for each run. The total number of loci for each run was 1207. The counts of loci under selection for each annotation in the tetraploid BUSTED run are emboldened as they were higher than in any of the diploid runs. The counts of each locus annotation under selection is emboldened for the tetraploid *Inga* run (bottom row).

**Ploidy**	**$\chi2$**	**df**	***P*-value**
Diploid set 1	4.5525	3	0.2077
Diploid set 2	8.3499	3	**0.03931***
Diploid set 3	5.099	3	0.1647
Diploid set 4	2.6054	3	0.4565
Diploid set 5	2.3295	3	0.5069
Diploid set 6	5.1536	3	0.1609
Tetraploids	4.0572	3	0.2554

Table S5: Chi-squared results testing the association between locus annotation and selection score, in terms of the number of loci in each category, for each ploidy level ('under selection' if false-discovery-rate-corrected P value <0.05). Columns indicate $\chi2$ value, degrees of freedom (df) and significance (P-value) for each $\chi2$ test. Tests showing a significant association between annotation and selection are emboldened and indicated by an asterisk (*).

	Annotation	$\chi 2$	df	*P*-value
Diploid set 1	Differentially expressed	13.832	1	**0.0001***
	Single copy phylo	84.162	1	**<2.2x10^{-16}***
	Defence chemistry	16.231	1	**5.606x10^{-5}***
	Misc	1.963	1	0.161
Diploid set 2	Differentially expressed	9	1	**0.0027***
	Single copy phylo	47.237	1	**6.291x10^{-12}***
	Defence chemistry	5.896	1	**0.015***
	Misc	3.24	1	0.072
Diploid set 3	Differentially expressed	5.907	1	**0.015***
	Single copy phylo	35.596	1	**2.428x10^{-9}***
	Defence chemistry	5.255	1	**0.0218***
	Misc	0	1	1
Diploid set 4	Differentially expressed	0.19	1	0.662
	Single copy phylo	2.635	1	0.104
	Defence chemistry	0.557	1	0.455
	Misc	0	1	1
Diploid set 5	Differentially expressed	4.609	1	**0.0318***
	Single copy phylo	41.217	1	**1.363x10^{-10}***
	Defence chemistry	9.614	1	**0.001***
	Misc	1.9631	1	0.161
Diploid set 6	Differentially expressed	0.524	1	0.469
	Single copy phylo	35.596	1	**2.428x10^{-9}***
	Defence chemistry	4.075	1	**0.043***
	Misc	3.24	1	0.072

Table S6: Chi-squared results testing the association between ploidy and selection score ('under selection' if false-discovery-rate-corrected P value <0.05), in terms of locus counts in each category, for each annotation. Columns indicate $\chi 2$ value, degrees of freedom (df) and significance (*P*-value) for each $\chi 2$ test. Tests showing a significant association between ploidy and selection are emboldened and indicated by an asterisk (*). Tests are grouped based on the diploid taxon set used for BUSTED analysis, each of which had ca. 28 taxa in order to match the number of polyploid taxa (n=29). In the chi-squared tests, the BUSTED results obtained for the tetraploid taxa served as the reference for comparison with each diploid taxon set (indicated by merged rows in the first column).
